# Systems Biology and Bile Acid Signalling in Microbiome-Host Interactions in the Cystic Fibrosis Lung

**DOI:** 10.3390/antibiotics10070766

**Published:** 2021-06-24

**Authors:** David F. Woods, Stephanie Flynn, Jose A. Caparrós-Martín, Stephen M. Stick, F. Jerry Reen, Fergal O’Gara

**Affiliations:** 1 BIOMERIT Research Centre, School of Microbiology, University College Cork, T12 YN60 Cork, Ireland; david.woods@ucc.ie (D.F.W.); 110318793@umail.ucc.ie (S.F.); 2 Human Microbiome Programme, School of Pharmacy and Biomedical Sciences, Curtin University, Perth 6102, Australia; jose.caparros-martin@telethonkids.org.au; 3 Curtin Health Innovation Research Institute (CHIRI), Curtin University, Perth 6102, Australia; 4 Wal-Yan Respiratory Research Centre, Telethon Kids Institute, Perth 6009, Australia; stephen.stick@health.wa.gov.au; 5 Telethon Kids Institute, The University of Western Australia, Perth 6009, Australia; 6 School of Biomedical Sciences, Faculty of Health and Medical Sciences, The University of Western Australia, Perth 6009, Australia; 7 Department of Respiratory Medicine and Sleep Medicine, Perth Children’s Hospital, Perth 6009, Australia; 8 School of Microbiology, University College Cork, T12 YN60 Cork, Ireland; 9 Synthesis and Solid State Pharmaceutical Centre, University College Cork, T12 YN60 Cork, Ireland

**Keywords:** cystic fibrosis, lung, microbiota, gastro-oesophageal reflux, chronic infection, pathogen, inflammation, bile acids, *Pseudomonas aeruginosa*, aspiration

## Abstract

The study of the respiratory microbiota has revealed that the lungs of healthy and diseased individuals harbour distinct microbial communities. Imbalances in these communities can contribute to the pathogenesis of lung disease. How these imbalances occur and establish is largely unknown. This review is focused on the genetically inherited condition of Cystic Fibrosis (CF). Understanding the microbial and host-related factors that govern the establishment of chronic CF lung inflammation and pathogen colonisation is essential. Specifically, dissecting the interplay in the inflammation–pathogen–host axis. Bile acids are important host derived and microbially modified signal molecules that have been detected in CF lungs. These bile acids are associated with inflammation and restructuring of the lung microbiota linked to chronicity. This community remodelling involves a switch in the lung microbiota from a high biodiversity/low pathogen state to a low biodiversity/pathogen-dominated state. Bile acids are particularly associated with the dominance of Proteobacterial pathogens. The ability of bile acids to impact directly on both the lung microbiota and the host response offers a unifying principle underpinning the pathogenesis of CF. The modulating role of bile acids in lung microbiota dysbiosis and inflammation could offer new potential targets for designing innovative therapeutic approaches for respiratory disease.

## 1. Introduction

Chronic respiratory diseases encompassing asthma, Chronic Obstructive Pulmonary Disease (COPD) and Cystic Fibrosis (CF) are a leading cause of death worldwide and are rapidly emerging as a global health problem [1]. Studies have revealed that the lungs of both healthy and diseased individuals harbour distinct microbiotas [2,3,4,5,6]. Perturbations of these communities are evident in chronic respiratory disease. This dysbiosis is characterised by a shift towards a low-diversity, pathogen-dominated microbiome with the infection process beginning as early as infancy. Novel clinical treatment strategies have recently been introduced (such as Orkambi) and these offer great benefits to CF patients. However, long-term chronic infections and heightened inflammation are still a major cause of concern [7,8]. Aggressive management of chronic infections can delay their progression, but cannot typically eradicate the problem, which has caused clinical emphasis to shift towards patient welfare. Therefore, elucidating the factors that are responsible for the switch to a chronic lifestyle in microbes is imperative. This review focuses on the Bile Acid (BA)–Lung microbiota axis in CF, an emerging issue with the potential to modulate the progression of chronic respiratory disease.

## 2. The Pathophysiology of Cystic Fibrosis

CF is an autosomal recessive genetic disease resulting from mutations in the Cystic Fibrosis Transmembrane Conductance Regulator (CFTR) gene [9]. These *CFTR* mutations are grouped into Classes 1–6 dependant on the type of mutation. These include nonsense mutations (Class 1), misfolded proteins (Class 2), gating mutations (Class 3), conduction mutations (Class 4), reduced protein quantity (Class 5) and unstable protein production (Class 6). With the advent of recent novel therapies, these have been regrouped to a more phenotypic classification of mutations, (a) where no CFTR protein is formed, (b) where there is a lack of CFTR protein at the cell surface and (c) where there is reduced or dysfunctional CFTR protein at the cell surface [10]. The *CFTR* gene encodes a channel that localises in the membrane of epithelial cells controlling ion transport [9]. Transport secretion is stimulated through CFTR by a number of factors, including cAMP signalling, phosphorylation, calcium-activated calmodulin and Bile Acids (BAs) [11,12]. Interestingly, the composite profile of serum BAs in a CF individual can be altered in comparison to a non-CF healthy individual [13]. This altered CF BA profile is also present in both the intestinal tract and skin and has been proposed as a biomarker for the diagnosis of CF [13,14,15].

According to the CF patient registry, CF affects approximately 70,000 people worldwide, particularly those of Caucasian descent [16]. Whilst it is known to be a multisystem disorder affecting the lungs, gastrointestinal tract, liver and the pancreas, the predominant cause of death in CF is associated with respiratory problems [17,18]. Up to 80% of CF deaths are caused by persistent chronic airway infections and inflammation [19]. The pathophysiology of CF is complex but can in part be attributed to alterations (dehydration and acidification) of the Airway Surface Liquid (ASL), leading to increased mucus viscosity on the lining of the epithelial cells within the lungs. This mucus facilitates the colonisation of the airways with bacteria, which cannot be removed from the lungs due to impaired mucociliary clearance [20,21]. Additionally, the heightened pro-inflammatory response to pathogens further exacerbates this problem. Specifically, the host cannot mount an effective and controlled immunity-driven clearance of the infection [22]. As a result, there is a perpetual cycle of infection and inflammation in the patient, which causes bronchiectasis and progressive lung decline [23,24]. It should be noted, however, that inflammation can occur independent of infection, which suggests that the mutation of the CFTR protein alone can contribute to inflammation [25]. Moreover, inflammation can persist post infection clearance, and is linked to bronchiectasis progression [26]. In addition to the well-defined pulmonary symptoms, a number of comorbidities have been described for CF, including but not limited to cystic fibrosis-related diabetes, cystic fibrosis-related liver disease and gastro-oesophageal reflux disease (GORD). These extra complications can further impact upon patient life expectancy and Quality of Life (QoL) [27].

### 2.1. Therapeutic Management of Cystic Fibrosis

Clinical management strategies currently focus on the consequences of the CFTR mutations. The classical strategies involve the prevention and control of pulmonary infections by reducing the viscosity and increasing clearance of mucus from the airways using chest physiotherapy and mucolytic therapies [28]. Additionally, the identification of infection-causing pathogens is critical, the goal of which is the eradication of pathogens with antimicrobials before the microbe can become established and persistently colonise the airways [29]. More recent approaches have targeted the CFTR protein in an attempt to restore protein functionality. This is achieved by the use of modulators, such as potentiators which are employed to maintain the CFTR protein in an open conformation. On the other hand, correctors aid the CFTR protein to adopt the correct confirmation, traffic to the cell surface and remain in place longer [30]. Currently these therapies include combinations of ivacaftor, lumacaftor, tezacaftor and elexacaftor. Widely used and approved CFTR modulators include, Kalydeco^®^ (ivacaftor), Orkambi^®^ (lumacaftor/ivacaftor), Symdeko^®^ (tezacaftor/ivacaftor) and Trikafta^®^ (elexacaftor/tezacaftor/ivacaftor). A number of CFTR modulators are also in development for the less common CFTR protein variants. Amplifiers are in development, which are a CFTR modulator that increases the amount of protein formed, as are stabilisers, which improve the quality of the CFTR channels [31,32]. Furthermore, gene editing therapeutics for CF are under development utilising CRISPR/Cas9 technologies [33]. In the treatment of CF patients, modulators have been shown to alter a wide range of key metabolomic signals such as phospholipids, bacteria associated metabolites and BAs [34,35].

Longitudinal studies with modulators demonstrate their practical beneficial outcomes for the treatment of CF. A CF cohort (*n* = 80) receiving Ivacaftor demonstrated a significant increase in FEV_1_% in a subset of the cohort (<12 years), and a nearly halving of the overall necessity for oral and intravenous antibiotic interventions (49% and 46% reductions, respectively) [36]. However, there was no significant reduction observed in hospitalisation, or in the chronicity status, of two of the dominant CF lung pathogens, *Pseudomonas aeruginosa* and *Staphylococcus aureus*. The halving of antibiotic treatments for exacerbations and the unfortunate maintenance of chronic *P. aeruginosa* infections is supported by other Ivacaftor CF treatment studies [37,38]. However, it is important to note that shorter term studies (6–12 months observation) of Ivacaftor treatments, do show a decrease in *P. aeruginosa* infections, with no change in *S. aureus* infections [8,39,40]. Gaining insights into the initial establishment and subsequent re-establishment (post initial modulator administration) of chronic lung microbial communities, could facilitate the maintenance of a healthy lung microbiota and function, and, therefore, increase QoL.

### 2.2. The Respiratory Microbiome

**Figure 1:**
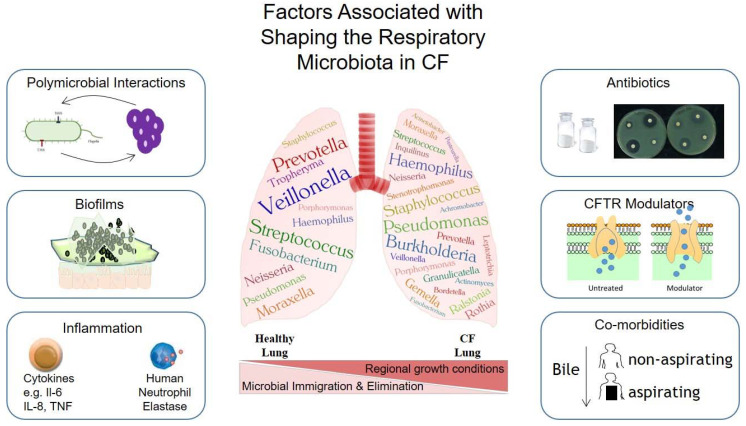
Summary of common microbiota associated with healthy vs. CF lungs. Commonly identified taxa are illustrated and the factors governing the dynamics of these communities are presented. Microbes, such as the anaerobes *Veillonella* and *Prevotella*, are routinely isolated from healthy lungs. While *Staphylococcus* and *Haemophilus* are known to be early colonisers of the lungs of paediatric patients with CF, *Pseudomonas* ultimately achieves a dominant position within the lung microbiota. The balance between microbial immigration and elimination has been described as a primary influence on the community structure of a healthy lung. In contrast, regional growth conditions within the lung microenvironment have been shown to drive population diversification. Summarized in the figure are factors that shape, model and remodel the lung microbiota. These include polymicrobial interactions which can influence the survival and infection acquisition, biofilm development that can increase the persistence of infections, inflammatory responses that can both directly and indirectly affect the bacteria within the lung, antibiotic use which can directly alter the lung microbiota as well as causing alterations in inflammation and the gut microbiome, CFTR modulators which can increase airway clearance and co-morbidities that can influence the lung microbiome directly and through interconnections such as the gut–lung axis.

The lungs of healthy individuals were previously considered to be a sterile environment due to the lack of detection of culture positive samples. However, several studies have now demonstrated that bacterial, fungal and viral communities are present in the lungs of both healthy and diseased individuals [2,4,6,41,42,43,44,45]. The respiratory tract has a surface area of ~75 m^2^ and it is estimated that a healthy lung is ventilated with 7000–20,000 L of microbially laden air per day [46,47,48]. These healthy lungs have a rich diverse microbial density of 10^3^–10^5^ CFU/g, with approximately 2.2 × 10^3^ bacterial genomes per cm^2^ [49,50]. Moreover, this microbially diverse healthy niche has a distinct bacterial signature. A healthy profile can be comprised of *Prevotella*, *Streptococcus*, *Neisseria*, *Veillonella*, *Corynebacterium *and* Fusobacterium*, accompanied by other dynamically changing colonisers (Figure 1) [2,50,51,52]. The nascent microbial sources of the lung microbiota can be of environmental, gut, skin, oral and pharyngeal origin [43,51,52]. Microaspiration has been proposed as a mechanism through which microbes can transition into the lungs [3], with Wu and colleagues reporting a potential immune-protective role [53]. Mitigation of susceptibility to *Streptococcus pneumoniae* was observed following episodic aspiration of oral commensals in a pre-clinical murine model [53]. The characterisation of a healthy lung microbiota is of central importance in further understanding the microbiological contribution to respiratory disease.

### 2.3. Infection Progression in CF Respiratory Diseases

The classical early paediatric pathogenic colonisers associated with CF lungs are *S. aureus* and *Haemophilus influenzae* [54]. These are superseded by *P*. *aeruginosa* in young adult patients, with upwards of 70% of adult CF airways being colonised by this pathogen [19,55]. Though still a major problem, reductions in chronic colonisation by *P. aeruginosa* have been observed with advances in clinical management strategies [56]. This has resulted in improvements in patient outcome and increased life expectancies [57,58]. Many CF lung infections are now considered polymicrobial and the interactions between members of the community can play an important role in the pathophysiology of the disease [59]. This has shifted our appreciation to the role of polymicrobial communities in infection and in the factors contributing to the restructuring of these communities throughout disease progression [60,61,62,63,64,65]. Moreover, the holobiont (defined as a host and its associated communities of microorganisms) concept is receiving more attention and, in particular, the nature of the interplay involved between the host and its associated microbiota. Together, the host and its microbiota that comprise a holobiont, has the potential to modulate the pathophysiology of a disease [66]. This hypothesis, highlights the importance of collectively treating the holobiont as a single entity rather than the microbiota and the host as distinct entities [67].

### 2.4. The Structure of the CF Lung Microbiota

While the exact nature of a stable healthy lung microbiota still needs to be conclusively defined, the microbiota in a CF lung is well characterised, with over 60 of the most commonly identified genera [68]. The most abundant genera are outlined in Figure 1, some of which are shared amongst healthy and diseased individuals [6,68]. Culture dependant clinical data from 49,886 CF patients from 38 countries revealed that the dominant isolated respiratory pathogens were *P. aeruginosa*, *Burkholderia cepacia* complex (Bcc) species, *S*. *aureus*, Methicillin-Resistant *S. aureus* (MRSA), Non-Tuberculous Mycobacteria (NTM), *Stenotrophomonas maltophilia*, *Achromobacter* species and *H. influenzae* [69]. Interestingly, using Next-Generation Sequencing (NGS) technologies, the presence of an abundance of facultative and obligative anaerobes, such as *Porphorymonas* and *Gemella* in the CF lung, has also been revealed. Such organisms had been largely undetected by conventional culturing practices [68,70]. However, these isolates are not unexpected, considering the anoxic environment created by the presence of a viscous mucus in the CF lung [71].

The lung microbiota in paediatric CF patients is quite diverse but, as patients transition to adulthood, there is a reduction in diversity. Often, the emergence of a single dominant pathogen, particularly *P. aeruginosa*, becomes evident [6,57,72]. This gradual loss of diversity has been correlated with a reduction in lung function [6,73]. However, the similarity in community composition between paediatric and adult microbiomes suggests that microbial dysbiosis becomes established early on in life and is maintained throughout disease progression [41]. An analogy of this pattern of behaviour is seen with the dysbiosis (and consequential alteration of the functional capabilities) of the gut microbiota for inflammatory diseases and diabetes, and also in the oral cavity microbiota dysbiosis associated with periodontitis [74,75]. This progressive loss of diversity and subsequent microbial dysbiosis is evident in CF with an increase in the dominance of pro-inflammatory pathogens, a key factor underpinning disease severity [76].

Significant spatial heterogeneity exists in the CF lung in which gradients of oxygen, pH and nutrients exist [77]. This heterogeneity has been shown to act as a significant selective pressure impacting on the community composition present throughout parts of the lungs [44,78,79]. Spatial uniformity is more evident in a healthy lung, suggesting a minimal influence from the environment to the structure of the bacterial communities present [2,42]. The rate of bacterial reproduction, immigration and elimination is an important determinant of the lung microbiota in health, with evidence demonstrating that these bacteria can enter healthy lungs via micro-aspiration [3,80].

This field of study has greatly benefited from the advent of next generation sequencing technologies. However, the lack of standardised approaches relating to sample collection (such as with sputum versus Bronchoalveolar Lavage Fluid (BALF)), method of DNA extraction and sequencing platform utilised has limited the ability to perform cross study comparisons [81]. The retrieval of lower airway samples that are free from contamination of the upper airways should also be a factor for consideration. This requires the inclusion of numerous processing controls for the accurate determination of the lung microbiota [2,80,82]. These issues must be continually addressed, and consistent practices developed and adapted, in order to strengthen the interpretation of data sets emerging from global studies.

### 2.5. Factors Shaping the CF Lung Microbiota

The composition of the lung microbiome has been shown to be determined by three primary factors; (1) the degree of microbial immigration, (2) the rate of microbial elimination and (3) the growth rate of the constituent members (Figure 1) [77]. The contribution and balance of each of these factors to the respiratory microbiota should be of primary consideration when studying differences that may exist between health and disease. Though there are few studies on a healthy lung microbiome, it is broadly accepted that bacteria within niches of the human holobiont are critical in the maintenance of a healthy functional immune system [50,83,84]. Diversity of microbiomes can be analysed using Alpha diversity (richness and evenness) and comparative Beta diversity [85]. The lower airways of healthy individuals have been shown to harbour diverse low abundance bacterial communities [2,3,42]. In contrast, the lower airways in respiratory disease consists of bacterial communities with shallow diversity and high abundance [4,5,57,61,72,86,87]. The differences in diversity and abundances between health and disease is potentially due to a higher bacterial burden and ineffective microbial clearance from the lungs affected by pulmonary disease, especially in CF patients [88]. These lower airway bacterial communities could be sourced from the upper airways, the environment and the gastrointestinal tract [41,89,90]. The reservoirs of bacteria can be spread via inhalation, micro-aspiration and direct mucosal extension [3,77].

Exacerbations, which are defined as an acute worsening of symptoms, do not display the classical features of a bacterial infection, such as an increase in the pathogen load or decrease in community diversity [91,92,93]. Interestingly, serum-based metabonomics research has suggested that pulmonary exacerbations in CF populations are associated with alterations in important signalling molecules, including BAs [94]. BAs are important signalling hormones that bind to nuclear receptors, and alterations in bile composition can influence the immune response [95]. Infections in CF lungs are considered polymicrobial in nature with exacerbations a consequence of microbial dysbiosis. This dysbiosis can occur as a consequence of alterations in signalling events [96,97]. Chronic infections with the key CF pathogen *P. aeruginosa*, have been demonstrated to drive this pulmonary dysbiosis, rather than the existence of prior dysbiosis facilitating the emergence of the pathogen [98]. This chronic infection and remodelling of the lung microbiome community is associated with a reduction in lung function and poorer clinical outcomes [98]. In addition to the impact of chronic *P. aeruginosa* colonisation on the structure of the lung microbiome, other factors, such as antibiotics, environmental cues, and inflammation, have been proposed to play a role in the shaping of the lung microbiota [72,99,100,101,102,103]. Studies have revealed both transient [5,102] and long-lasting [72,99] effects on the lung microbiome by the aforementioned factors. It is clear, however, that the frequent disruptions to these microbial communities are associated with increased morbidity and mortality [91,104].

### 2.6. The Role of Bile Acids (BAs) in Shaping the CF Lung Microbiome

The host factor that has recently emerged as a driver of alterations in the temporal dynamics of the lung microbiota in people with CF is BAs. Synthesised in the liver from cholesterol, these host factors are a grouping of amphipathic steroid molecules. They can be categorised as either primary or secondary BAs, and are detected in conjugated and unconjugated forms [105]. A diverse gut microbiome is responsible for the deconjugation and formation of secondary BAs [106]. The roles of BAs include digestion, vitamin absorption, solubilisation of cholesterol, bile flow induction, bacteriostasis and, importantly, signal molecules [107]. Although primarily studied as a host factor associated with the liver and intestinal system, BAs have recently been detected in the lungs (both in BALF and sputum samples) of CF patients [108,109,110,111].

Several studies have reported that BAs detected in the lungs correlate with alterations in the CF lung microbiota. The stratification of paediatric patient cohorts based on detected BA concentrations in sputum revealed a significant reduction in the biodiversity and richness of the microbiota within the lungs [110,112]. While this was a small-scale study, the emergence of dominant Proteobacterial pathogens, such as *Pseudomonas*, *Stenotrophomonas* and *Ralstonia*, in some cases representing up to 98% of the microbiome of bile positive samples, was notable [110,112]. Clinical parameters, such as age, gender and antibiotic treatments, were not associated with the shift in the microbiome in these samples [110]. Patients in whose sputum samples the levels of BAs were below the level of detection exhibited a richer biodiversity when compared with the BA positive samples. This microbial diversity was underpinned by species associated with a healthy non-CF lung, such as the anaerobes, *Veillonella* and *Prevotella* [5]. Subsequently, a longitudinal analysis of BALF samples from the Australian Respiratory Early Surveillance Team for Cystic Fibrosis (AREST CF) biobank in Australia revealed a link between the detection of BAs and the presence of specific bacterial groups, rather than with single microbial ecotypes per se [113]. The presence of BAs in the lungs is associated with pathogen abundance and continuous exposure to BAs in the lung indicates that an ecological process of differential microbial succession may be operating [113,114]. Patients whose samples were continuously BA positive exhibited distinct co-occurrent microbial profiles when compared to patients where BA detection was either intermittent or absent. Specifically, high levels of BAs were shown to correlate with a reduction in oral bacteria (*Rothia*, *Streptococcus*), and the acquisition of emergent opportunistic pathogens (*Stenotrophomonas*, *Ralstonia*, *Moraxella*) [113]. The consistency of the correlation with respect to BA levels and pathogen colonisation between sputum and BALF studies is notable, and it suggests a lower respiratory tract dimension to this microbe-BA interaction. BAs in the lungs have also been shown to correlate with increased colonisation by *P. aeruginosa* post-transplantation, with the induction of IL-8-mediated neutrophilic airway inflammation, implicated as a potential mechanism [115].

Although the presence of BAs in lung sputum and BALF samples has been demonstrated by several research groups, the mechanism through which BAs accumulate in the lungs remains to be determined. In situ de novo BA biosynthesis or routing through the circulatory system have both been proposed as sources of BAs present in the lungs [116,117]. However, the detection of significant levels of glycine-conjugated primary BA GCA would appear to contradict the latter hypothesis, due to the fact that distal intestinal absorption is compromised in CF patients [118]. Indeed, the presence of conjugated primary and secondary BAs in the lungs would be consistent with a liver and/or hepatic/gall bladder source for these host factors. The inadvertent introduction of BAs into lung sputum or BALF samples caused by bleeding during collection or by pre-existing structural lung damage has also been considered, although the evidence would suggest that neither is a likely cause [114]. There is, however, growing evidence that gastric contents which are refluxed and aspirated into the lungs during periodic episodes of Gastro-oesophageal reflux (GOR)/Duodenogastroesophageal reflux (DGER) could be a primary source of pulmonary bile [110,111,119,120]. Evidence for the gastric source of BAs is supported by the effectiveness of Nissen Fundoplication surgery in the control of reflux and potentially in the prevention of aspiration [121,122]. Moreover, this practice has also shown concomitant improvements in lung function [121,123,124,125]. The rate of bile aspiration associated with GOR is estimated to be as high as 80% in CF patients [108].

Gastro-oesophageal reflux disease (GORD) has been described as a major comorbidity in numerous respiratory conditions (Table 1) [126,127,128,129,130,131], and it has been shown to correlate with increased levels of BAs in CF lungs [132]. The incidence of GORD in people living with CF can be as high as 40% in paediatric patients, rising to over 80% in adults [111,133,134,135]. Even this may be an under estimation due to limitations regarding diagnosis and the prevalence of silent GORD [136,137]. GORD has also been shown to correlate with increased lung disease severity [138,139]. The correlation between GORD-derived reflux, pulmonary aspiration, and increased lung damage extends to a wide range of respiratory diseases (Table 1) [140]. These include advanced lung damage following lung transplantation [141,142], ventilator-associated pneumonia [143], Barrett’s oesophagus and oesophageal adenocarcinoma [144] and Bile Acid Pneumonia in neonates [117]. El-Serag and Sonnenberg demonstrated that patients with erosive oesophagitis, a sign of advanced GORD, had increased incidence of pulmonary fibrosis, chronic bronchitis or COPD in a case control study of more than 200,000 patients [145].

In CF, GORD is proposed to be due to a variety of factors including (a) an increased number of transient lower oesophageal sphincter relaxations, (b) increased intra-abdominal pressure and (c) delayed gastric emptying [146,147]. Therapeutics for the treatment of GORD in CF patients include, Proton Pump Inhibitors (PPI), gastroprokinetic agents, and Histamine-2 Receptor Antagonists (H2RA) [139,148]. Stretta Radiofrequency (SRF), a non-invasive treatment similar to Nissen Fundoplication has also been proposed as a treatment option [139]. This procedure strengthens the barrier between the stomach and the oesophagus [149,150]. It is worth noting that where these treatment options target the acidic nature of reflux rather than preventing the reflux action itself, the transition of BAs into the lungs would not necessarily be affected. It follows that the impact of BAs on inflammation and microbiome dynamics in the lungs of affected patients would, therefore, proceed uninhibited. This underscores the need to identify the aspect of GORD that underpins the correlations with lung damage and pathogen colonisation.

### 2.7. Bile Acids (BAs) Trigger a Switch towards a Chronic Biofilm Lifestyle in Respiratory Pathogens

**Figure 2:**
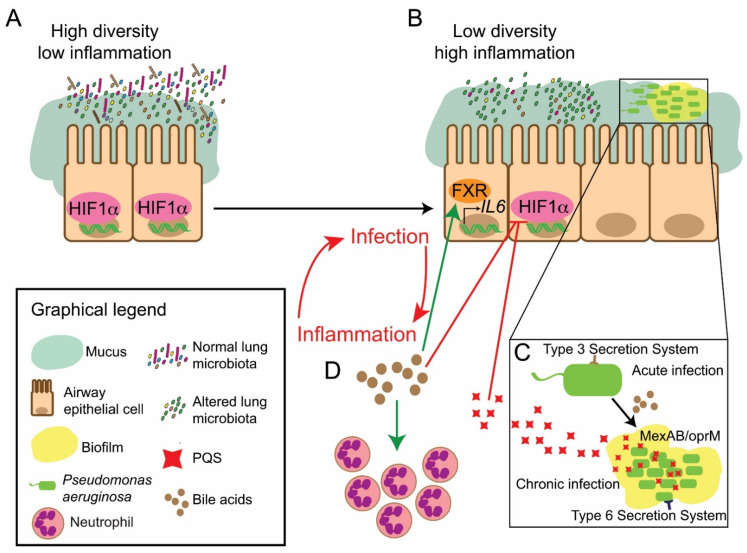
Overview of the proposed BA-microbiota axis within the CF holobiont. (**A**,**B**) The accumulation of BAs within the lungs of patients with CF as a consequence of aspiration leads to changes in the structure of the lung microbiota. This results in a transition from an (**A**) high diversity stable community to a (**B**) pathogen-dominated low diversity population. (**B**–**D**) Modulation of host signalling through the HIF-1 transcription factor by BAs and bile-induced *P. aeruginosa* derived Pseudomonas Quinolone Signal (PQS) serves to further promote dysregulated inflammation in CF patients. The impact of BAs on the behaviour of the key CF-associated pathogen *P. aeruginosa* (**C**) leads to a switch towards a chronic antibiotic tolerant biofilm lifestyle, with increases in PQS, the MexAB RND efflux pump and the Type Six Secretion System (T6SS), in parallel with a reduction in T3SS. (**B**–**D**) There is a dual impact of BAs on the lung microbiota, promoting chronicity and the inflammatory response. BAs also cause an FXR-dependent induction of pro-inflammatory cytokines and, consequently, the chemoattraction of neutrophils. This evidence underscores the unifying principle of BAs as a major host factor promoting the progression of chronic respiratory disease.

The link between BAs and changes in the lung microbiota is the result of the direct actions of BAs in microbes in the lung, it would be expected that these effects could be demonstrated in vitro. Indeed, bile has been shown to exert a direct influence on the virulence, pathogenesis, and antibiotic tolerance of members of the human microbiota, including the ESKAPE pathogens [106,204,205]. Previously, studies investigating bile–bacterial signalling were largely focused on enteric organisms and their capacity for bile tolerance and resistance [206,207]. The more recent finding that bile and individual BAs can modulate the behaviour of key respiratory pathogens has further supported a role for this host factor in the modulation of respiratory disease. Bile and BAs are capable of triggering the key CF associated pathogen *P. aeruginosa* to adopt a chronic biofilm lifestyle [112,208]. The ability of the pathogen to switch to this biofilm lifestyle renders it refractory to antibiotic therapy [209,210]. Moreover, bile promotes increased antibiotic tolerance to clinically relevant antibiotics, such as colistin, polymyxin B and erythromycin [112]. Additionally, bile has been shown to induce a programmed transcriptional response facilitating this lifestyle change. This transition is underpinned by a repression of acute virulence systems, such as type-three secretion, phenazine production and swarming motility, induction of chronic virulence systems, such as type-six secretion, quorum sensing and biofilm formation, and the reprogramming of central metabolism in the cell (Figure 2) [112]. This adaptive response to bile could underpin this organism’s capacity to outcompete other members of the lung microbiota. Furthermore, the ability of *P. aeruginosa* to thrive in the acid-suppressed stomach of patients receiving proton pump inhibitors for the treatment of GORD is of concern due to suggestions of the existence of an aero-digestive microbiota [89,211]. This is of particular relevance, as bacteria may have prior exposure to bile with the resultant aspiration allowing for the introduction of pre-adapted isolates into the lungs [89,212]. These pre-adapted isolates may, therefore, have an additional competitive advantage over residential members of the lung microbiome.

The impact of bile is not restricted to *P. aeruginosa*. Bile also induces biofilm formation in several other key respiratory pathogens, such as *Burkholderia cepacia* complex and *Acinetobacter baumanii*, while repressing biofilm in others such as typed strains of *S. aureus* and *S. maltophilia* [208]. In contrast to, yet consistent with the hypothesis of population heterogeneity within the CF lung, clinical isolates of *S. aureus* and *S. maltophilia* adopted a biofilm lifestyle as observed in other respiratory pathogens [112,213]. Bile positively induces the quorum-sensing molecule, Pseudomonas Quinolone Signal (PQS) (as well as homoserine lactones) [208]. PQS is capable of controlling inter-species communication. This could further impact the lung microbiome community, thus showing a possible indirect signalling regulation mediated by bile [214,215]. Moreover, PQS, and its biological precursor 4-hydroxy-2-heptylquinoline (HHQ), suppress biofilm formation in co-colonising bacterial and fungal CF pathogens, such as *Aspergillus fumigatus* and *Candida albicans* [214,216,217]. Induction of these compounds in the presence of bile may, therefore, influence the interactions between *P. aeruginosa* and competing organisms in the BA positive lungs of CF patients. Collectively, these studies support a key signalling role for BAs in modulating the pathophysiology of respiratory disease.

### 2.8. Bile Acids (BAs) Elicit a Pro-Inflammatory Response in Respiratory Disease

In addition to the correlation between BAs and chronic respiratory colonisation, the accumulation of BAs in the lungs has also been linked to airway inflammation. Detected concentrations of BAs vary and have been shown to be capable of inducing lung damage and inflammation in cultured lung epithelial cells [218,219]. Moreover, the presence of BAs in the lungs have been associated with impaired innate immunity and reduced levels of important pulmonary surfactants [220,221,222,223]. Increased levels of alveolar neutrophils [108] and interleukin-8 (IL-8) [113,115,224] were reported in patients with elevated levels of BAs. Furthermore, BA aspiration has also been associated with increased Tumour Necrosis Factor alpha (TNF-α) in a rodent model of chronic aspiration [225]. The molecular mechanisms underpinning BA induced changes in the inflammatory response are as yet undefined, although mechanistic insights into the role of BAs as host signals continue to emerge [226,227]. While a role for pro-inflammatory pathogens in instigating inflammation has been reported, some studies suggest that airway inflammation can occur in infancy [228]. Within a CF paediatric cohort, longitudinal exposure to BAs upregulated the important pro-inflammatory markers of IL-1β, IL-6, IL-8 and neutrophil percentage [113]. It is, therefore, possible that the impact of BA aspiration on lung inflammation is a result of the activation of inflammatory markers, as well as the indirect tandem promotion of pro-inflammatory pathogens, such as *P. aeruginosa*. A similar BA-mediated pathomechanism has been proposed to explain the development of liver injury in cholestasis [229]. BAs also have direct effects on CF human lung epithelial cells causing cytotoxicity [218]. However, it is important to note that different BAs have varying degrees of cytotoxicity at the same physiologically relevant concentrations. The monohydroxylated secondary BA, LCA, had the most cytotoxic effect of nearly 100%. The dihydroxylated BAs, CDCA and DCA had less of a cytotoxic effect of approximately 20%, whereas the trihydroxylated primary BA, Cholic acid (CA) showed <10% cytotoxicity on the lung cells [218]. These results are crucial, as they focus the relevance and importance of profiling the spectrum of human BAs and gives possible therapeutic gut and lung target options.

As well as promoting the colonisation and chronicity of pro-inflammatory pathogens, BAs can affect the host inflammatory response. Neutrophil dominated inflammation is a characteristic pathophysiology of CF, and BAs have been shown to mediate this process in intestinal cells [227]. D’Ovidio and colleagues reported a link between the aspiration of gastro-oesophageal refluxate and the development of Bronchiolitis Obliterans Syndrome (BOS) following lung transplants. Increased levels of both neutrophil elastase and IL-8 in BALF were associated with elevated levels of BAs. The same pattern of inflammatory marker induction in the presence of BAs has also been described in patients with ventilator-assisted pneumonia [143], where the primary BA, chenodeoxycholic acid (CDCA), was associated with elevated levels of IL-8.

Analysing a clinical paediatric CF cohort (*n* = 88) identified BA profiles in randomly selected bronchoalveolar lavage fluid (BALF) [114]. A paediatric cohort was selected so to investigate the initial triggering factors that modulate the transition to chronic microbial infection and inflammation. BALF was assessed in an attempt to mitigate oral contributions to the sampling [230]. Chronic inflammatory markers have been linked to the progressive development of bronchiectasis, lung function decline and respiratory failure [231,232]. Analysing the paediatric CF BALF demonstrated an association between the presence of BAs and an alteration in the levels of inflammatory markers, specifically with elevated levels of Interleukin 1 beta (IL-1β) and IL-6 [114]. Moreover, BA profiles were shown to be a meaningful predictor for the progression of lung disease. The induction of these pro-inflammatory cytokines (IL-1β and IL-6) has been linked to the severity of other respiratory diseases including Severe Acute Respiratory Syndrome Coronavirus 2 (SARS-CoV-2/COVID-19) [233,234,235]. The host-derived factor, bile, could contribute to the progression of respiratory disease in CF patients, as well as further highlighting the importance of the gut–lung axis [236]. Further evidence in support of the link between bile and lung disease in individuals with CF was provided by a longitudinal study undertaken on BALF sampled from clinically stable children with CF [113]. The temporal study demonstrated that BAs in the lungs were associated with alterations in the expression of inflammatory markers over time. Furthermore, the presence of BAs in the lungs was also associated with a less desirable disease trajectory in terms of lung structural damage [114]. However, while evidence of a correlation is supported from several studies, this does not infer causation. Further work in that regard is required to elucidate the mechanistic basis for these strong correlations.

BAs have been demonstrated to act as signalling molecules through the activation of dedicated receptors, such as the nuclear receptor, Farnesoid X Receptor (FXR) and the membrane-bound receptor, Takeda-G protein Receptor 5 (TGR5). In addition, BAs can also activate other receptors, such as the pregnane X receptor which has been shown to be a mediator of gut dysbiosis in response to cholesterol-lowering therapies [226]. In vitro reports have described how BAs modulate the production of pro-inflammatory markers, including FXR-dependent elevated levels of IL-6 production in lung epithelial cells [112,237,238]. This suggests that bile aspiration could drive the dysregulation of the inflammatory response. Moreover, the BA receptor FXR, has been demonstrated to be expressed in human airway epithelial cell lines [12] and involved in a wide range of respiratory diseases, such as COPD, asthma and idiopathic pulmonary fibrosis [239].

BAs are also capable of modulating the host immune response by targeting the Hypoxia-Inducible Factor (HIF)-1 transcription factor [218]. This transcription factor is important in the mounting of an effective host response to infection [240]. BAs destabilise the HIF-1α subunit in an FXR and TGR-5 independent manner [112]. HIF-1 has previously been shown to be essential for the resolution of acute inflammation [241,242]. Destabilisation could, therefore, underpin chronic inflammatory pathophysiology associated with elevated BAs in patients with respiratory disease. Interestingly, BAs induce PQS, which has also been shown to destabilise HIF-1α in lung epithelial cells [218] suggesting a dual targeting of this key host transcriptional checkpoint in the *P. aeruginosa* infected BA positive lung environments. Indeed, PQS is produced at high levels in the lungs of *P. aeruginosa* positive paediatric patients with CF, particularly in isolates obtained from patients under the age of three [243]. Destabilisation of HIF-1α in these patients may further contribute to the ability of *P. aeruginosa* to avoid host clearance [244].

## 3. Future Perspectives

The range of therapeutic options available for the clinical management of CF has increased significantly in recent years with the successful introduction of modulators [245,246]. A wide range of CFTR modulators are in development for the less common CFTR protein variants. Further CF therapies are currently being developed. These include gene editing, mRNA repair, premature termination read-through drugs, protein repair, amplifiers, pharmacological chaperones, proteostasis regulators, and novel potentiators, as well as gene transfer therapeutics for the management of CF are also in development [31,32,33,247]. However, despite progress, many significant obstacles continue to delay the development of these strategies as clinical treatments for CF. Pre-clinical trials have shown evidence of only modest improvements in FEV1, but not in the QoL of affected patients [248]. Infections, exacerbations and inflammation continue to be a persistent problem for people living with CF. Chronicity and lung function decline treatments must continue to be a strong focus in these CF individuals to improve their QoL and reduce morbidity. The identification of factors and signals promoting the emergence of chronic pathogens must be a fundamental priority in the design and eventual implementation of novel treatment regimens.

An additional factor that needs to be considered in the context of precision medicine is the importance of cell-type in relation to CF. A comparative single cell resolution analysis from a multi-institute consortium has provided a molecular atlas of the proximal airway epithelium [249]. The authors demonstrated that, while all major human airway epithelial cell types (basal, secretory, and ciliated) are common between a healthy and CF lung, there is a disease-dependant difference within the CF lung-cell composition. The proximal airway of patients with CF undergoing transplantation were shown to have an overabundance of epithelial cells transitioned to specialised ciliated and secretory cells subtypes and a decrease in cycling basal cells when compared with healthy patients [249]. In a separate study, Okuda and colleagues reported that secretory cells dominate CFTR expression and function in human airway superficial epithelia [250]. Investigation into the signalling behind this transition and apparent selection is warranted from a systems biology perspective in order to further understand the mechanism underlying this process and its contribution to CF pathophysiology. A wide array of host and/or microbially derived signalling molecules could be responsible, as could the microbiome with its inherent biotransformational potential. There is already evidence within the intestine that BA act as a signal for selective increases in cell subtype densities, renewal, and proliferation [251,252,253].

A comprehensive understanding of the impact of bile on the community structure and composition of the lung microbiome is essential in the design of increasingly effective treatment plans for CF. An overview of the BA–microbiota–inflammation axis in the CF holobiont is outlined in Figure 2. The ultimate goal is early intervention for the prevention of chronic infections and inflammation, possibly signalled by bile induced lung damage. BA profiling has the potential to be developed as a biomarker in CF patients most at risk of developing disease associated microbiome signatures [113,114]. Therefore, the development of point of care devices for routine use in clinical settings represents a unique opportunity in the clinical care of CF patients. In addition to the early detection of BAs in the lungs, there is an additional need for the early detection of *P. aeruginosa* infection and other bile-responsive respiratory pathogens, which have the capacity for a programmed response to bile [110,113,114]. The commencement of strict eradication regimens in order to prevent the establishment of pathogenic microbiomes is correlated with improved patient health outcomes [58,254].

The utilisation of the macrolide antibiotic, azithromycin, has been linked to improvements in lung function in lung transplant recipients [255]. As well as its antimicrobial properties, azithromycin has multiple immunomodulatory effects, including the inhibition of pro-inflammatory cytokines, induction of regulatory functions of macrophages, inhibition of neutrophil influx, and alterations in autophagy [256]. The use of azithromycin has been linked to a reduction in GOR and BA aspiration in lung transplant recipients [257]. Thus, it may provide a potential option for reducing GORD (and BA reflux) leading to improved lung function. Azithromycin is routinely used in CF patients and has been linked to a lower risk of infections by common CF respiratory pathogens [258]. The administration of azithromycin in respiratory patients, for which aspiration represents a significant clinical issue, should be further investigated in light of these beneficial effects [257].

Further profiling and analysis of the complex interactions within the lung could give rise to inhaled lung probiotic and prebiotic treatments to outcompete and restore a dynamic homeostasis within this niche [96]. Moreover, now that there is a stronger link established between the lungs and the gut, conventional gut therapies and pre/pro-biotics could aid in restoration of lung function and reduce inflammation [259,260]. Inhaled BA sequestrants and/or degradation enzymes to titrate out free BAs and silence their effects within the lungs is also worth considering as an innovative therapeutic strategy in the clinical management of CF. A combination therapy of antibiotics and BA sequestrants may represent a potential future therapeutic strategy.

## 4. Conclusions

In this review we have summarised the following key issues to help understand the development and progression of CF disease: (1) the dual impact that BAs exert on both the host and the microbiota, thus providing a unified principle underpinning the progression of CF disease; (2) BAs have been associated with a reduction in microbial diversity and the promotion of pathogen dominance; (3) reviewed the direct regulatory effect of bile on key CF respiratory pathogens; (4) detailed the underpinning mechanism through which BAs modulate a variety of human proteins, such as CFTR, HIF-1 and FXR, possibly facilitating an understanding of how BAs exacerbate airway pathology. The presence of BAs in the lungs has been associated with a number of respiratory diseases and conditions other than CF, including Chronic Lung Allograft Dysfunction (CLAD), BOS and bacterial colonisation [115,223,261]. Therefore, the issue of BA aspiration may extend beyond CF and its relevance as a key host signal modulating lung disease should be further examined. Further investigations are required to decipher the molecular mechanisms underpinning the lung microbiota community remodelling in the presence of bile. Respiratory conditions continue to be amongst the leading causes of death, further compounded by the recent lack of new antibiotic development. The identification of BAs as a modulator of lung microbiota dysbiosis and inflammation is promising. Specifically, it presents a new drug target option to be evaluated for the effective management of these conditions.

## Figures and Tables

**Table 1:** Literature review of the association of gastro-oesophageal reflux and aspiration with a range of respiratory conditions.

Respiratory Condition	Clinical Presentation	Reference
Cystic Fibrosis	Reflux	[128,130,133,151,152,153,154,155,156,157]
	Reflux/aspiration	[108,109,111,119,126,158,159]
COPD	Reflux	[160,161,162,163,164,165,166,167,168,169,170,171]
	Reflux/aspiration	[172,173]
Asthma	Reflux	[174,175,176,177,178,179,180,181,182,183]
	Reflux/aspiration	[184]
Chronic cough	Reflux	[185,186,187,188,189,190,191,192]
	Reflux/aspiration	[193,194,195]
Idiopathic pulmonary fibrosis	Reflux	[196,197,198,199,200,201]
	Reflux/aspiration	[202,203]
